# Comparative Transcriptome Analysis Reveals Related Regulatory Mechanisms of Androgenic Gland in* Eriocheir sinensis*

**DOI:** 10.1155/2017/4956216

**Published:** 2017-11-09

**Authors:** Chunpeng Fu, Qifan Zeng, Fajun Li, Huicui Wang, Jian Sun, Hui Wang

**Affiliations:** ^1^College of Animal Science and Technology, Shandong Agricultural University, Tai'an 271018, China; ^2^Weifang University of Science and Technology, Weifang 262700, China; ^3^The Fish Molecular Genetics and Biotechnology Laboratory, School of Fisheries, Aquaculture, and Aquatic Sciences and Program of Cell and Molecular Biosciences, Auburn University, Auburn, AL 36849, USA

## Abstract

Chinese mitten crab* (Eriocheir sinensis)* is one of the most commercially important aquaculture species in China. The androgenic gland (AG) of crustaceans plays pivotal roles in the regulation of male differentiation and in maintaining the male sexual characteristics. In order to reveal related mechanisms in AG, we compared transcriptomes of AG between proliferation and secretion phase. A total of 72,000 unigenes and 4,027 differentially expressed genes were obtained. Gene ontology enrichment analysis indicated that biological processes and metabolic pathways related to protein synthesis and secretion such as transcription, translation, and signal transduction were significantly enriched. Critical genes such as* IAG, SXL, TRA-2, SRY, FTZ-F1, FOXL2*, and* FEM-1* were identified and potentially involved in maintaining the testis development and spermatogenesis. Ribosomes pathway revealed the cause of insulin-like androgenic gland hormone secretion increase. Three insulin-like receptors were thought to be associated with growth and spermatogenesis. In the neuroactive ligand-receptor interaction pathway, the expression of octopamine receptor, 5-HT receptor 1, and melatonin receptor was significantly changed, which revealed the key regulation mechanism of aggressive and mating behavior of males. Comparative transcriptome analysis provided new insights into the genome-wide molecular mechanisms of AG development and the regulatory mechanisms of male development.

## 1. Introduction

Chinese mitten crab* (Eriocheir sinensis)* is one of the most important aquaculture species, which is widely distributed in freshwater and low-salinity estuarine regions in China [1, 2]. Its culture under facility conditions started in the early 1980s and the annual output has increased during the past decade [3]. In 2014, the domestic total production was more than 750,000 tons for aquaculture only. The male mitten crabs grow faster and are more aggressive than females in the current traditional culture system, which may result in high mortality of the females and thus influence the production of the crab. It is obvious that this culture of all-male or all-female populations would be more economically advantageous to crab aquaculture industry [4, 5].

It was reported that monosex culture has been achieved through sex reversal mediated by steroid manipulations and has been practiced in fish aquaculture [6]. Unlike fish, sexual differentiation in crustaceans is uniquely regulated by the male-specific androgenic gland (AG), which has been detected in male crustaceans. It plays a pivotal role in the regulation of male differentiation and in maintaining the male sexual characteristics [4, 7–13]. So far, the insulin-like androgenic gland hormone (IAG) was identified in many commercially important crustacean species [14–18].* IAG* silencing in* Macrobrachium rosenbergii* proved to be useful in obtaining a full and functional sex reversal species [19], leading to the production of monosex populations. So far, AG cDNA libraries and transcriptome datasets serve as a basis for further studies [20, 21], but the molecular mechanism pertaining to the related regulatory mechanisms of AG in* E. sinensis* is still unclear.

Transcriptome is a total set of transcripts, mRNA, and noncoding RNA, in one or a population of cells during a specific developmental stage or in response to a particular physiological condition using high-throughput technology [22]. It is the connection between genome genetic information and proteomics biological function [23]. In nonstandard model organisms where genomic resources are lacking, such as a fully sequenced genome, obtaining a transcriptome is an effective way to evaluate gene expression and perform comparative studies at the whole genome level [24]. Comparative transcriptome analysis provides the foundation for gene structure and function research and determines when genes are expressed and how they are regulated [25]. Now, it is being widely applied to elucidate genetic factors conferring economically significant traits in cultured crustacean species [26–28].

In the current study, we generated AG transcriptomes using cDNA prepared from mRNAs isolated at the proliferation phase (PP) and secretion phase (SP) in* E. sinensis* and performed a comparative analysis. The objectives of the present study were to elaborate the genetic expression change between PP and SP during AG development and to reveal the molecular regulation mechanisms of stimulating spermatogenesis and maintaining male characteristics.

## 2. Materials and Methods

### 2.1. Preparation of Samples, cDNA Library Construction, and Sequencing

All of the specimens were obtained from a farm in Ganyu, Jiangsu province, China, in October 2015. The PP and SP AG were identified and characterized under the microscope according to those described by Qiu et al. [29]. Three replicated samples were prepared for PP and SP AG, respectively. Fifty individuals were included in each sample.

Total RNA was isolated using TRIzol reagent according to the manufacturer's instructions. The isolated RNA was treated with RNase-free DNase I (Sangon, Shanghai, China) to eliminate possible genomic DNA contamination. Equal quantities of total RNA from three replicate samples were mixed to prepare the pooled RNA sample for RNA-Seq. RNA purity was checked using the Nano Photometer® spectrophotometer (IMPLEN, CA, USA) and RNA concentration was measured using Qubit® RNA Assay Kit in Qubit® 2.0 Fluorometer (Life Technologies, CA, USA). RNA integrity was determined using the RNA Nano 6000 Assay Kit of the Bioanalyzer 2100 system (Agilent Technologies, CA, USA).

The transcriptome libraries for sequencing were generated using the VAHTS™ mRNA-Seq v2 Library Prep Kit for Illumina® (Vazyme Biotech Co., Ltd., Nanjing, China) following the manufacturer's recommendations. The clustering of the indexed samples was carried out using the VAHTS RNA Adapters of Illumina (Vazyme Biotech Co., Ltd., Nanjing, China) according to the manufacturer's instructions. After clustering, the libraries were sequenced on Illumina HiSeq X Ten platform, with 150 bp pair-end reads produced.

### 2.2. Transcriptome Assembly

Raw images were transformed into raw reads by base calling using CASAVA (Version 1.8.2). Clean reads were obtained by removing reads with adapters, reads in which unknown bases were more than 5%, and low-quality reads (the percentage of low-quality bases was over 50% in a read, and we defined the low-quality base to be the base whose sequencing quality was no more than 10). At the same time, Q20, Q30, and GC content of the clean data were calculated.

### 2.3. Gene Annotation

Gene functional annotation was performed by sequence comparison with public databases. The unigenes of the de novo assembly were searched against the NCBI nonredundant (NR), Swiss-Prot, KEGG, and COG protein databases by using BLASTX with a cut-off *E* value of 10^−5^.

### 2.4. Expression Annotation and Differentially Expressed Genes (DEGs) Analysis

FPKM (fragments per kilobase per transcript per million mapped reads) method was performed to quantify the expression of two expression profiles [30]. The False Discovery Rate (FDR) method was applied in hypothesis testing and multiple hypothesis testing to calibrate significant levels and eliminate influence of random fluctuations and errors [31]. At the same time, according to the gene expression level (FPKM value), differentially expressed multiples in different samples were calculated. The differentially expressed genes (DEGs) were defined as FDR ≤ 0.001 and no less than 2-fold.

### 2.5. GO and KEGG Enrichment Analysis of DEGs

First, we mapped differentially expressed genes to each term of the GO database (http://www.geneontology.org/) and calculated the gene number of each term, and thus we got a GO function gene list and gene number statistics. Then, we used hypergeometric inspection, compared with the entire genome background, to find out significantly enriched GO entries in the differentially expressed genes.

KEGG pathway was the unit for pathway significant enrichment analysis. The *P* value calculation formula of the hypothesis test was similar to that of GO function significant enrichment analysis. After multiple testing corrections, we chose the pathway with *Q*-value ≤ 0.05 as a significant enrichment pathway in the differentially expressed genes.

### 2.6. Real-Time Quantitative PCR (qRT-PCR) Verification

Six candidate DEGs were randomly selected for validation by QPCR. Total RNA from independent samples of PP and SP was extracted separately and reverse-transcribed using PrimeScript® RT Reagent Kit with gDNA Eraser (TaKaRa, Dalian, China). The SYBR Green RT PCR assay was carried out in an ABI PRISM 7300 Sequence Detection System (Applied Biosystems). Six pairs of gene-specific primers (*IAG*,* IR*,* OA receptor*,* 5-HT receptor 1*,* Tra-2*, and* FTZ-F1*; Table 1) designed using Premier Primer 5 were used to amplify the partial cDNA gene sequences, respectively. Three biological replicates for each sample and three technical replicates were performed. The *β*-actin from* E. sinensis* was chosen as a reference gene for internal standardization [32]. PCR was carried out in a total volume of 10 *μ*l, containing 5 *μ*l of 2x SYBR Premix Ex Taq (TaKaRa, China), 0.2 *μ*l of 50x ROX reference dye, 2 *μ*l of the diluted cDNA mix, 0.2 *μ*l of each primer (10 *μ*M), and 2.4 *μ*l of sterile distilled H_2_O. The PCR program was 95°C for 5 min, followed by 40 cycles of 95°C for 5 s and 60°C for 31 s. To confirm that only one PCR product was amplified and detected, dissociation curve analysis of amplification products was performed at the end of each PCR reaction. After the PCR program, data were analyzed with ABI7300 SDS software (Applied Biosystems). The relative expression level was calculated using the 2^−ΔΔCt^ method. The results were subjected to one-way analysis of variance (one-way ANOVA) using SPSS 16.0, and *P* values less than 0.05 were considered statistically significant.

## 3. Results

### 3.1. Assembly Results and Unigene Functional Annotation

21.392 and 55.29 *μ*g RNA were obtained, respectively, whose qualities meet the requirements of the sequencing and building libraries. A total of 49,925,104 and 49,975,260 raw reads were obtained from the PP and SP transcriptomes. All of the raw reads were deposited into the Sequence Read Archive (SRA) database (https://www.ncbi.nlm.nih.gov/Traces/sra/) under accession numbers SAMN06204640 and SAMN06204641. After removing adaptors and low-quality reads, a total of 47,335,722 and 46,323,850 clean reads were obtained in each of the profiles, respectively. After de novo assembly, 72,000 unigenes were obtained by paired-end method of Trinity and TGICL clustering. Among them, a total of 30,576 unigenes were significantly matched with NR database.

**Figure 1:**
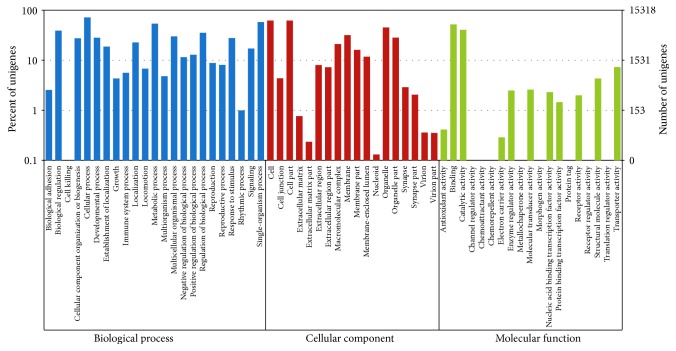
All-unigene GO classification. Each annotated sequence was assigned at least one GO term. GO terms at the 2nd level were plotted here, and in this ontology, “biological process,” “cellular component,” and “molecular function” are categorized independently.

Based on GO analysis, 15,318 unigenes were annotated and divided into three categories: “biological process,” “cellular component,” and “molecular function” (Figure 1). The number of matched GO terms per unigene varied from 1 to 143. More than 89.1% of unigenes could be assigned to more than one GO term, with the majority of unigenes mapped to 2 to 7 GO terms. Based on GO analysis, three major functional groups were divided into 60 subcategories, including the dominant subcategories, cellular process (10955), cell (9486), cell part (9471), single-organism process (8860), metabolic process (8220), and binding (7958).

For the eggNOG analysis, a total of 14,125 unigenes were assigned to 25 function categories (Figure S1 in Supplementary Material, available online at https://doi.org/10.1155/2017/4956216). Among them, 6,996 unigenes were assigned to general function prediction only, representing the largest group, followed by translation, ribosomal structure, and biogenesis (5065) and cell cycle control, cell division, and chromosome partitioning (3995). KEGG analysis revealed that 24,691 unigenes were assigned to six categories comprised of 258 metabolic or signaling pathways. The most prominent KEGG pathways were metabolic pathways (2683, 10.87%), regulation of actin cytoskeleton (1768, 7.16%), amoebiasis (1635, 6.62%),* Vibrio cholerae* infection (1409, 5.71%), focal adhesion (1225, 4.96%), RNA transport (1060, 4.29%),* Salmonella* infection (1048, 4.24%), adherens junction (958, 3.88%), bacterial invasion of epithelial cells (938, 3.8%), and chemokine signaling pathway (889, 3.6%).

### 3.2. Differentially Expressed Genes (DEGs) Analysis between the Two Developmental Stages

**Figure 2:**
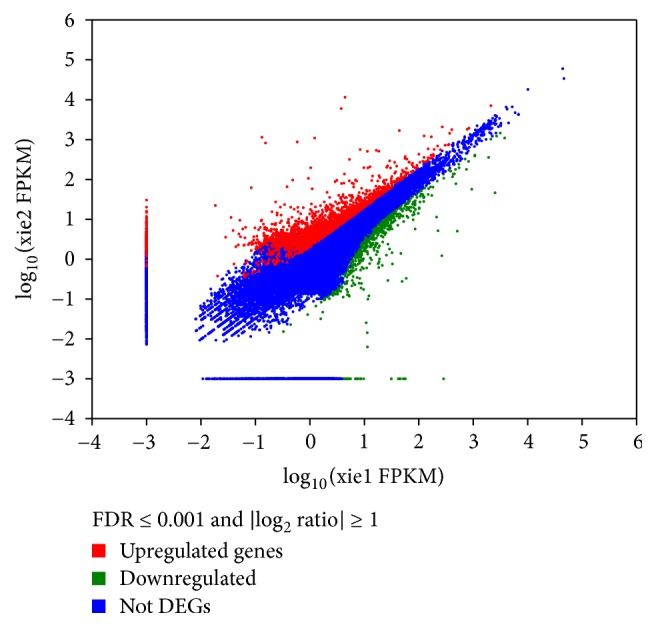
Expression level of PP versus SP. For each unigene, the ratio of expression levels (PP versus SP) was plotted against the log error rate. The horizontal line indicates the significance threshold (FDR < 0.05), and the vertical lines indicate the twofold change threshold. Upregulated genes are shown with red dots, and downregulated genes are shown with green dots. Non-differentially expressed genes are shown with blue dots.

After comparison with fold change threshold value and FDR test, there were 4,027 genes from the total 70,915 genes (5.68% of the genes) with a significant difference (*P* < 0.05) between PP and SP stages, including 2,220 upregulated genes and 1,807 downregulated genes. The distribution of the significant changes of 70,915 unigenes was illustrated in a scatter diagram (Figure 2), where the statistical significance of each unigene was plotted against fold change. Functional distribution of these DEGs was further analyzed by GO and KEGG enrichment, respectively.

To gain further insights into the molecular mechanisms of attractive quality formation during AG development between PP and SP, gene ontology enrichment analysis was performed to determine which kind of GO term DEGs were mainly enriched in. Finally, 2,345 DEGs were annotated to 3,588 GO terms. The functions of 854 DEGs with higher expression in SP than in PP AG were assigned to biological process, cellular component, and molecular function. In biological process, SRP-dependent cotranslational protein targeting to membrane (41 DEGs, GO: 0006614), nuclear-transcribed mRNA catabolic process, nonsense-mediated decay (42 DEGs, GO: 0000184), cotranslational protein targeting to membrane (41 DEGs, GO: 0006613), protein targeting to ER (41 DEGs, GO: 0045047), and establishment of protein localization to endoplasmic reticulum (41 DEGs, GO: 0072599) were the most prominent terms. Cytosolic ribosome (44 DEGs, GO: 0022626) was the most prominent term within the cellular component followed by ribosomal subunit (46 DEGs, GO: 0044391), cytosolic large ribosomal subunit (27 DEGs, GO: 0022625), ribosome (49 DEGs, GO: 0005840), and extracellular exosome (96 DEGs, GO: 0070062). In molecular function, most of the annotated unique sequences were assigned to phosphatidylserine binding (13 DEGs, GO: 0001786), structural constituent of ribosome (48 DEGs, GO: 0003735), and modified amino acid binding (14 DEGs, GO: 0072341).

KEGG annotation is the process of mapping genes of interest to the metabolic pathways. In our study, 1,863 DEGs were mapped to 243 KEGG pathways. The significantly changed pathways included ribosome (46 DEGs, ko03010), cytosolic DNA-sensing pathway (21 DEGs, ko04623), neuroactive ligand interaction (52 DEGs, ko04080), RNA polymerase (36 DEGs, ko03020), pancreatic secretion (34 DEGs, ko04972), ECM-receptor interaction (53 DEGs, ko04512), and cell adhesion molecules (25 DEGs, ko04514), all of which should be the key metabolic networks leading to cell proliferation, secretion, and signal transduction in AG.

### 3.3. qRT-PCR Verification

**Figure 3:**
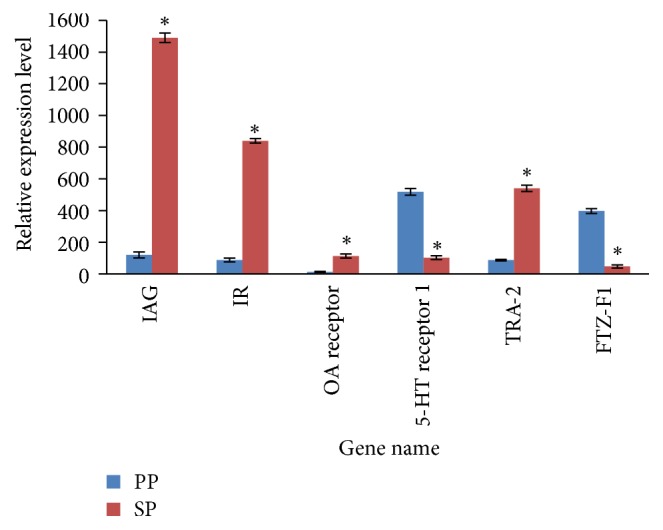
QPCR analysis of 6 selected DEGs. *x*-axis: the 6 differentially expressed unigenes; *y*-axis: relative expression level of each unigene. *β*-Actin is an internal control gene. *∗* indicates a significantly different expression (*P* < 0.05).

The six DEGs were selected to verify the results of the RNA-Seq analysis by qRT-PCR using different RNAs from those used for RNA-Seq (Table 2). The results showed that the expression patterns of* IAG*,* IR*,* OA receptor*,* Tra-2*,* 5-HT receptor 1*, and* FTZ-F1* all agreed well between RNA-Seq and qRT-PCR, which confirmed the data obtained from high-throughput sequencing (Figure 3).

## 4. Discussion

Although the mechanism of sex differentiation in crustaceans has yet to be defined, the androgenic gland (AG) is thought to be the exclusive organ that produces the androgenic hormone, which induces male sexual development. The AG system serves as a unique biological system for studying endocrine regulation of sex differentiation in decapods. GO and pathway analyses of DEGs will be helpful in the discovery of novel genes that play key roles in maintaining male characters and related behaviors during AG development.

### 4.1. Candidate DEGs Involved in Male Development

*IAG* was found to be expressed in the AG of male crustaceans, which belongs to the insulin superfamily of proteins and was considered as the key regulator of male sex differentiation [5, 12].* IAG* is responsible for the development and continuation of male primary and secondary sexual characteristics, inhibiting the synthesis of Vg and stimulation of spermatogenesis. We found that a unigene from the AG transcriptome sequencing result has 44.2% and 42.4% identity with* IAG* of* Callinectes sapidus* and* Scylla paramamosain*, respectively. Further analysis found that the length of the gene sequence is 1392 bp and ORF length is 456 bp. The amino acid sequence alignment indicated that the novel gene had common conservative amino acids with* Callinectes sapidus*,* Scylla paramamosain*, and so forth (Figure 5), which proved the novel gene is* IAG *of* E. sinensis*.

*Transformer-2 (Tra-2)* gene plays a key role in the regulatory hierarchy of sexual differentiation in somatic tissues and in the germline of* Drosophila melanogaster*.* Sex*-*lethal (Sxl)* plays a complicated and important role in embryogenesis, metamorphosis, somatic sexual development, and sex differentiation in insects and its highest expression levels were observed in testis and hepatopancreas [32, 33]. In our transcriptome database, we identified four Tra-2 isoforms designated as* Estra-2a*,* Estra-2b*,* Estra-2c*, and* Estra-2d*. Together with sex-lethal* (Sxl)* and double sex* (dsx)*, most of ortholog genes in* “Sxl*-*Tra*/*Tra-2*-*Dsx*/*Fru”* pathway are detected. In the process of male differentiation, the expression of* IAG* and* Tra-2* was significantly upregulated (Table 2), whereas the expression change of* Sxl* and* Dsx* was not significant, which illustrated that* IAG *and* Tra-2* may play a more important role in promoting the development of testis and stimulating male related behaviors.

DEAD box RNA helicase 20* (DDX20)*, forkhead transcription factor 2* (FOXL2)*, and fushi tarazu factor 1* (FTZ-F1)* were investigated in the regulation of vitellogenesis (VTG).* FOXL2* negatively regulates the VTG synthesis at developmental stage.* FOXL2* downregulates VTG's expression by binding* DDX20* in the regulation of follicular cell apoptosis and repress the synthesis of VTG when bound with* FTZ-F1* at the mature stage [18, 34, 35]. According to our research, during the process of testis development,* FOXL2* had no obvious change, and* FTZ-F1* gene was downregulated. Therefore,* FTZ-F1* may negatively regulate the testis development. Furthermore, cytochrome* P450* upregulated significantly in the SP indicates that the mechanism of cytochrome P450 regulating gonad development in fish and crustaceans may be completely different.

In* C. elegans*,* Fem-1*, encoding an ankyrin repeat protein, is a component of the signal transduction pathway controlling sex determination [36]. There were three members in the* Fem-1* gene family (designated as* Fem-1a*,* Fem-1b*, and* Fem-1c*) in our transcriptome database, which were homologs of the nematode* Fem*-1 protein [21]. It is reported that* Fem-1* might function in early sex determination and late gonad development in the crab [37]. However, in the present study, the four homologs of* Fem-1* are not significantly upregulated in the secretory phase of AG.

Research about spermatogenesis and the structure of the androgenic gland in* Eriocheir sinensis* showed that there was a close relationship between the androgenic gland and spermatogenesis. There was no spermatid when the androgenic gland was in the primary development phase [13]. With the AG development, a great number of primary and secondary spermatocytes appeared in the seminiferous tubules. Later, the sperms matured and were released when the androgenic gland was in the secretion phase.

The ubiquitin-proteasome pathway influences many biological processes, including cell degradation and protein homeostasis maintenance. Many studies have shown that ubiquitin may be relevant to heat shock proteins [38, 39]. For instance, heat shock cognate 70 kDa proteins are synthesized in haploid cells during spermatogenesis and are mainly activated at the spermatid stage [40], which may help ubiquitin and target nonrepairable proteins to the proteasome [41]. In the present study, a ubiquitin gene was found to be significantly upregulated in SP (Table 3). The serpin (serine proteinase inhibitor) family is reported to be exclusively expressed in the* rat cauda* epididymis and is upregulated, induced by androgens, and is secreted into the lumen to cover the sperm head. It can transform immotile spermatids into motile and fertilization-competent spermatozoa [42]. Here, we identified two upregulated unigenes, CL4939.Contig2 and CL5743.Contig1, that were annotated as serine proteinase inhibitors.

In crustaceans, cathepsin A functioned in the innate immune system [43]. The purified cathepsin A proteins of* E. sinensis* can effectively digest the spermatophore wall [44]. Cathepsin B was reported to control the developmental processes in insects and other arthropods. Cathepsin D is necessary for the formation of the yolk. Cathepsin L regulates the development of the ovary in many species including* L. vannamei*. Cathepsins A, B, C, D, F, and L were found in AG transcriptome. Cathepsin A was significantly upregulated while cathepsin D was significantly downregulated in SP AG, implying that these two types of cathepsins may play essential roles in the spermatogenesis process.

These results indicated that sex-related genes, such as* IAG, SXL, TRA-2, SRY, FTZ-F1, FOXL2*, and* cytochrome p450*, might play important roles in maintaining the male physiology and morphology of* Eriocheir sinensis*.* Ubiquitin*,* serpin*,* cathepsin A*, and* cathepsin D* genes may have a direct influence on spermatogenesis.

### 4.2. Ribosome Pathway Analysis

Ribosomes, the workplace for protein biosynthesis, are directly associated with translation, cell growth, cell cycle, localization, and cell proliferation [45]. In our present study, the ribosome is the most prominent pathway (*P* value: 1.130046*e* − 12). In 2011, the first complete atomic structure of eukaryotic 80S ribosome from the yeast* Saccharomyces cerevisiae* was obtained by crystallography. The assembly model of 80S ribosomes requires the joining of 40S and 60S subunits [46], which reveals the architecture of eukaryote-specific elements and their interaction with the universally conserved core. During translation initiation, the 40S subunit is interactive with eukaryotic translation initiation factor 1 (eIF1) and 60S subunit is in complex with eIF6. The core of the 60S subunit is formed by the 28S ribosomal RNA, which contributes the active site of the ribosome [47, 48].

**Figure 4:**
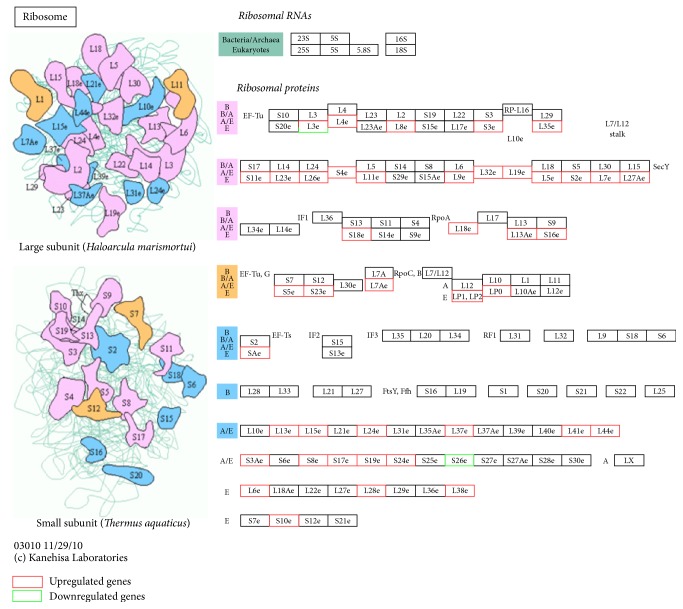
Ribosome pathway. The pathway is based on a KEGG pathway analysis. The upregulated and downregulated genes are labeled by red and green, respectively.

**Figure 5:**
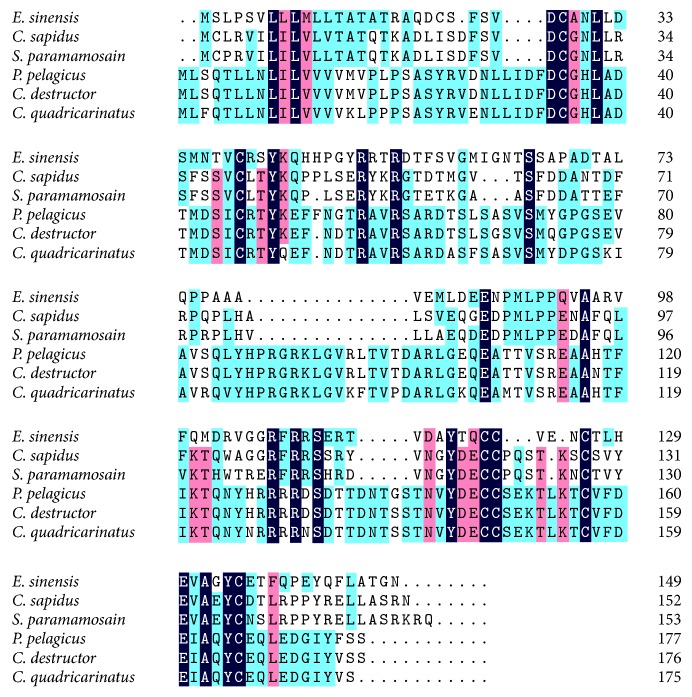
Amino acid sequences alignment of* Eriocheir sinensis* IAG with other crustacean IAGs. Species names are abbreviated at the left and represent* Callinectes sapidus* (HM594945.1),* Scylla paramamosain* (JQ681748.1),* Portunus pelagicus* (HM459854.1),* Cherax destructor* (EU18788.1), and* Cherax quadricarinatus* (DQ851163.1).

Comparative transcriptomic analysis showed that a total of 44 DEGs, including 40s and 60s ribosomal protein genes, are significantly upregulated; only ribosomal protein L3 and 40S ribosomal protein S26-like are significantly downregulated in the secretion phase of AG (Figure 4). Histological observation has shown that the secretion phase of AG has developed more rough endoplasmic reticulum and abundant free ribosome, while the synthesis of material is more active to maintain the secretion function compared with the proliferation phase [49]. The developmental cytology of the androgenic gland cells can be distinctly divided into proliferation phase and secretion phase [49]. Cells of the newly formed parts of the androgenic gland are generally located on the surface of the subterminal portion of the ejaculatory duct near the base of the penis. And the structure of the gland changed greatly during testis development cycle. Later, cells of androgenic gland increase in size, becoming multinucleated and vacuolated [50].

The results indicated that the androgenic gland development is a highly coordinated developmental program. The ribosome related genes might be the key elements in relation to cell growth and secretion during AG development.

### 4.3. Insulin Receptor and Signal Transduction

Sexual differentiation and maintenance of masculinity in crustaceans have been suggested to be regulated by* IAG*. However, downstream elements involved in the signaling cascade remained unknown. O. Sharabi and his colleges performed long-term RNA interference (RNAi) silencing in young male prawns to investigate the function of* M. rosenbergii* insulin receptor* (Mr-IR)*. They found that the silencing of* Mr-IR* advanced the appearance of a male-specific secondary trait but had no effect on growth. The most noted effects of* Mr-IR*'s silencing were hypertrophy of the AG and the associated increased production of* Mr-IAG*. These results suggested a role of* Mr-IR* in the regulation of the AG and implied sexual differentiation in crustaceans involving more than a single* Mr-IAG* receptor, which emphasized the complexity of sexual differentiation and maintenance [51]. The pivotal protein in any insulin family-based signaling pathway is the insulin receptor that is responsible for mediating the signal carried by insulin-like peptides (ILPs) from the intercellular to the intracellular environment. In the present study, a total of three* Es-IRs *were identified for the first time in the AG comprehensive transcriptomic library.

The insulin-like superfamily includes two major subgroups, ILPs and the insulin-like growth factors (IGFs) (Chandler et al., 2015). The role of the insulin pathway is very diverse and includes not only glucose homeostasis but also regulation of growth, longevity, and reproduction [52]. IAG is the sole ILP widely found in decapod crustaceans thus far, with the exception of a* Drosophila* ILP7 ortholog of unknown function, identified in a spiny lobster species [53]. IR and IGF1R are transmembrane receptors activated by insulin and insulin-like growth factor-1 (IGF-1). During the process of the AG development, 25 differentially expressed genes in the insulin signaling pathway were identified, among which were insulin receptor, translation initiation factor, factor facilitated glucose transporter, phosphorylase kinase gamma subunit, fatty acid synthase, and hormone-sensitive lipase genes. Unigene10231 and CL1931, mapped to the reference of insulin receptor cDNAs, are key genes in the insulin signaling pathway in* E. sinensis*.

So far,* IAG* has been shown to be a key regulator of reproductive processes such as sexual differentiation, spermatogenesis, and sexual shifts in crustaceans. Insulin receptors are pivotal players in insulin signaling pathway (Figure S2), which mediate insulin and ILP signaling [54]. However, apart from characterization of the hormone itself, little information regarding other elements of the signal transduction pathway is available. Unigene10231 is also identified as an insulin-like growth factor-1 receptor in progesterone-mediated oocyte maturation and oocyte meiosis pathways (Figure S3). Oocyte development can be inhibited by the androgenic glands when cultured in vitro. Unigene10231 was significantly downregulated in SP AG and participated in progesterone-mediated oocyte maturation and oocyte meiosis pathways. We can infer that Unigene10231 may indirectly regulate spermatogenesis and male secondary sexual characteristic development. In addition, whether CL1931 participates in sexual differentiation is unclear, which still needs further study.

### 4.4. Neuroactive Ligand-Receptor Interaction

It is reported that a large amount of melatonin can lead to testicular atrophy in hamsters [55]. While melatonin is produced in invertebrates and has influences on the physiology and behavior, little is known about its mechanisms of action [56]. The eyestalks of crustaceans contain the optic lobes and the x-organ/sinus gland, a neuropeptide-secreting neurohemal structure that is analogous to the vertebrate hypothalamus-pituitary system. The concentration of melatonin is high in the eyestalk of crustaceans [57]. The removal of eyestalk induces the hypertrophy of androgenic gland and precocious spermatogenesis in nonbreeding adult males [58].

Melatonin acts mainly via high-affinity receptors coupled to heterotrimeric guanine nucleotide-binding regulatory proteins (G proteins). Three high-affinity melatonin receptor subtypes have been cloned so far and classified as MT1a (also called MT1), MT1b (or MT2), and MT1c [59]. The MT1a receptor subtype is believed to mediate major neurobiological functions. In our study, melatonin receptor gene (Unigene23893_All) was significantly downregulated. This means that melatonin has a negative effect on the development of AG and thus influences the development of testis in* E. sinensis*.

Dopamine, octopamine, and serotonin are important signal molecules in the nervous systems of all multicellular animals. They are thought to exert hormonal control in a variety of behavioral contexts, including feeding behavior and aggression [60, 61]. Beltz et al. found that the biogenic amines 5-hydroxytryptamine (5-HT) and octopamine (OA) play a significant role in determining mating behavior in the lobster* H. americanus* [62, 63].

Serotonin receptors, also known as 5-hydroxytryptamine receptor or 5-HT receptor, are a group of G protein-coupled receptors and ligand-gated ion channels found in the central and peripheral nervous systems. They mediate both excitatory and inhibitory neurotransmission. The serotonin receptors are activated by the neurotransmitter serotonin, which acts as their natural ligand [64]. The serotonin receptors modulate the release of many neurotransmitters and influence various biological and neurological processes such as aggression, anxiety, appetite, cognition, learning, memory, mood, nausea, sleep, and thermoregulation. And great differences exist in 5-HT receptor subtypes; some of them play inhibitory roles, while others play exciting roles. In our study, the 5-HT receptor 1 was significantly downregulated in SP AG, implying that we found that the 5-HT receptor 1 gene negatively regulates the development of AG.

In crickets, injection of the OA agonist chlordimeform causes normally submissive losers of fights to reengage in fighting faster than sham injected animals [65]. Likewise, in honeybees, injection of two OA agonists, XAMI and DCDM, biases the likelihood of aggressive display toward nonnestmates over nestmates [66]. Hoyer et al. found that aggression is almost abolished in mutant males lacking octopamine in* Drosophila* by using software to eliminate confounding effects [67]. The expression of octopamine receptor was significantly upregulated in PP AG, indicating that the OA receptor positively regulated the aggression and mating behavior of* E. sinensis*.

Barki and colleagues removed AG from juvenile intersex crayfish and observed that in the adult stage they did not fight with other males and did not initiate mating behaviors with females [68]. We can see that AG has played a decisive role in male mating and aggressive behavior in* E. sinensis*. We found that the expression of octopamine receptor was significantly upregulated while 5-hydroxytryptamine receptor 1 was significantly downregulated in neuroactive ligand-receptor interaction pathway in SP AG (Figure S4). The function implementation of 5-HT and OA was regulated indirectly by AG. Neuroactive ligand-receptor interaction pathway analysis revealed the key regulation mechanism of melatonin, 5-HT, and OA, which have laid the foundation for further research.

## 5. Conclusions

This is the first comprehensive transcriptome dataset report for the AG of proliferation phase and secretion phase in* E. sinensis*. A total of 72,000 unigenes and 4,027 DEGs were obtained. Comparative transcriptomic analysis showed that* IAG, SXL, TRA-2, SRY, FTZ-F1, FOXL2,* and* cytochrome* p450 might play important roles in maintaining the male physiology and morphology.* Ubiquitin*,* serpin*,* cathepsin A*, and* cathepsin D* genes may have a direct influence on spermatogenesis. Ribosomes are the most prominent pathway and their related genes might be the key elements in relation to cell growth and secretion. In insulin-like receptor signaling pathway, Unigene10231 may indirectly regulate spermatogenesis and male secondary sexual characteristic development, and whether CL1931 participates in sexual differentiation still needs further study. In neuroactive ligand-receptor interaction pathway, octopamine receptor was significantly upregulated and 5-HT receptor 1 and melatonin receptor were significantly downregulated. This revealed the key regulation mechanism in male mating and aggressive behavior. Our comparative transcriptomic analysis provides new insights into the genome-wide molecular mechanisms of AG development and the regulatory mechanisms of male important traits of* E. sinensis*.

## Supplementary Material

Figure S1: COG function classification of Eriocheir sinensis androgenic gland transcriptome. Figure S2: Differentially regulated genes involved in insulin signaling pathway. The up-regulated and down-regulated genes are labeled in red and green, respectively. Figure S3: Differentially regulated genes involved in progesterone-mediated oocyte maturation. The up-regulated and down-regulated genes are labeled in red and green, respectively.

## Figures and Tables

**Table 1:** Oligonucleotide primers designed for QPCR of six candidate DEGs.

Unigene ID	Annotation	Regulation	Forward (5′-3′)	Reverse (5′-3′)
CL7578.Contig2_All	*IAG*	Up	CGGTTACAGGAGGACTCGAG	TGAGTGTAGGCGTCAACAGT
CL1931.Contig3_All	*IR*	Up	CCTCCTTCACCTTCCTCTCG	CTGTTGGAGATGGTGATGCG
Unigene3466_All	*OA receptor*	Up	TTCACGCGGAACTCACAAAG	CGATCCTACACCTTTGCTGC
Unigene35855_All	*5-HT receptor*	Down	GCTTCCCATTCGTCTCACAC	CGTGGTGGAGGTGAACAATG
Unigene24209_All	*TRA-2*	Up	CAGGTACGAGGCAAAATCCG	ATGTCCATGGCTCAACTGGA
Unigene21119_All	*FTZ-F1*	Up	TTGATCATCCGGGAGCTTGT	GCAAGTCAGACCAAGAGTGC

**Table 2:** qRT-PCR validation and comparative analysis with RNA-Seq data.

Gene	qRT-PCR ΔCT (mean ± SE)		qRT-PCR expression ratio 2^−ΔΔCT^	RNA-Seq log_2_(fold change)
	SP	PP	SP versus PP	SP versus PP
*IAG*	5.04 ± 0.07	11.71 ± 0.61	12.42 (*P* = 0.009)	1.75^*∗*^
*IR*	6.11 ± 0.32	9.14 ± 1.62	9.06 (*P* = 0.010)	1.01^*∗*^
*OA receptor*	8.64 ± 0.19	11.83 ± 1.46	8.69 (*P* = 0.027)	1.46^*∗*^
*5-HT receptor 1*	8.84 ± 0.22	6.45 ± 0.07	5.03 (*P* = −0.041)	−1.70^*∗*^
*TRA-2*	9.07 ± 0.16	10.83 ± 0.18	6.21 (*P* = 0.038)	1.08^*∗*^
*FTZ-F1*	9.81 ± 0.75	7.02 ± 0.56	8.45 (*P* = 0.032)	−1.33^*∗*^

^*∗*^Statistically significant difference is detected (*P* < 0.05).

**Table 3:** Male-related genes significantly up- or downregulated in PP AG.

Genes names	Unigenes	Description	Matched species	*e*-value	Fold change	Padj
I*AG*	CL7578.Contig2_All	Insulin-like androgenic gland factor	*Callinectes sapidus*	3.00*E* − 23	1.74772186825242	0

Tra-2	Unigene24209_All	Transformer-2 protein	*Daphnia pulex*	3.30*E* − 06	1.07968107604142	0

*Cytochrome* p450	CL5545.Contig1_All	Cytochrome P450	*Bemisia tabaci*	1.00*E* − 77	1.60200191612237	8.10*E* − 15
	CL4233.Contig1_All	Cytochrome P450 6BQ13	*Tribolium castaneum*	3.00*E* − 84	1.0661533430132	2.97*E* − 18
	Unigene20205_All	Cytochrome P450, family 4	*Drosophila persimilis*	7.00*E* − 54	1.04183996235728	7.49*E* − 05
	CL5412.Contig1_All	Cytochrome P450 4C	*Portunus trituberculatus*	2.00*E* − 173	1.04049844062959	1.19*E* − 07

*FTZ-F1*	Unigene21119_All	Fushi tarazu-factor 1	*Metapenaeus ensis*	0	−1.33163470883895	1.42*E* − 42

*Cathepsin D*	Unigene36945_All	Cathepsin D-like protein	*Homarus americanus*	4.00*E* − 63	−6.65470447032436	3.36*E* − 29
	Unigene32077_All	Cathepsin D-like protein	*Homarus americanus*	1.00*E* − 80	−4.0162234149032	1.61*E* − 36

SRY	Unigene46072_All	Sex-determining region Y protein	*Mus musculus*	7.00*E* − 09	11.2821038351649	6.96*E* − 05

*Serpin*	CL4939.Contig2_All	Serine proteinase inhibitor	*Procambarus clarkii*	1.00*E* − 27	1.33970323770525	2.03*E* − 06
	CL5743.Contig1_All	Serine proteinase inhibitor 2	*Portunus trituberculatus*	3.00*E* − 134	1.15750596081486	2.53*E* − 05

*Cathepsin A*	Unigene14462_All	Cathepsin A	*Eriocheir sinensis*	0	1.258868580610811	0

*Ubiquitin*	CL6912.Contig1_All	E3 ubiquitin-protein ligase	*Acromyrmex echinatior*	1.00*E* − 99	1.70587428423238	2.05*E* − 08

A positive fold change means gene expression increased, and negative fold change means gene expression decreased.
