# Supplementary figures and images for: Comparative Transcriptome Analysis Reveals Related Regulatory Mechanisms of Androgenic Gland in Eriocheir sinensis
Source: Biomed Res Int. 2017 Nov 9;2017:4956216. doi: 10.1155/2017/4956216 (PMC5700504; doi:10.1155/2017/4956216)

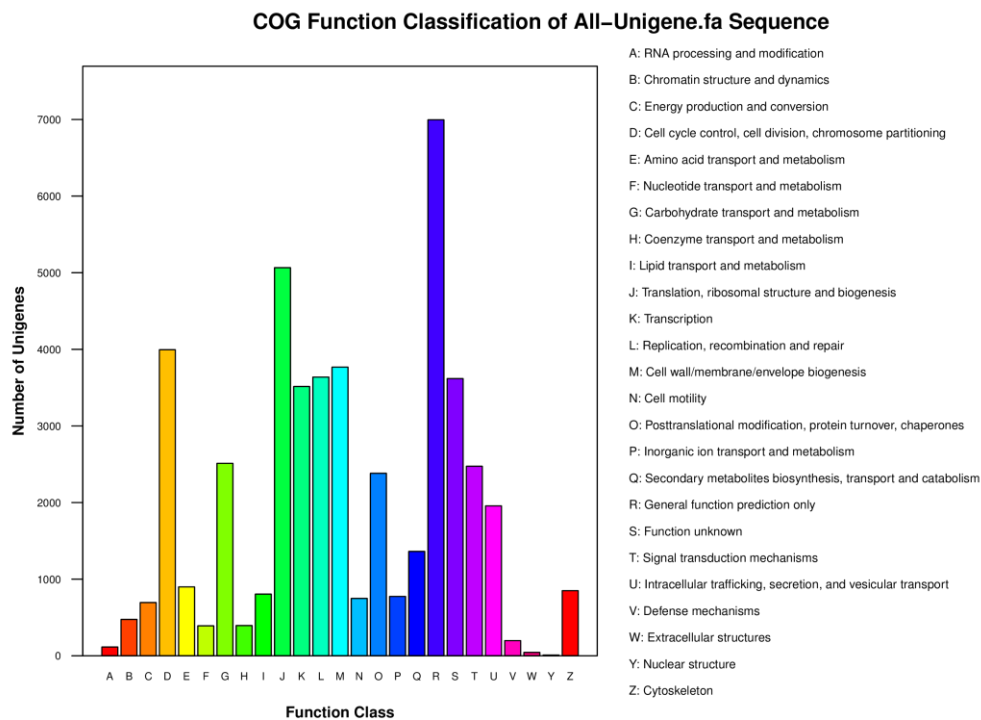

883

884

885

886

887

888

889

890

891

892

893

894

895

896

897

898

899

900

901

902

903

904

905

906

907

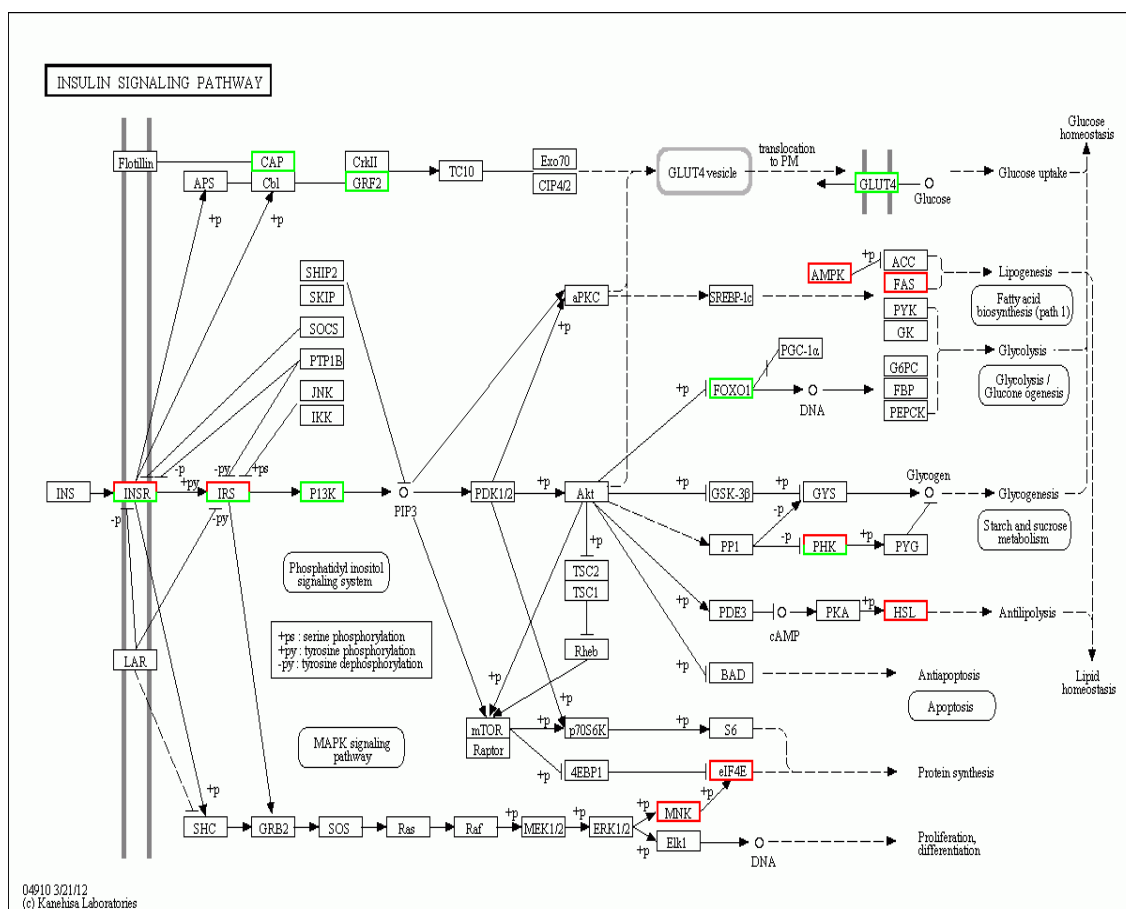

S3 Fig.

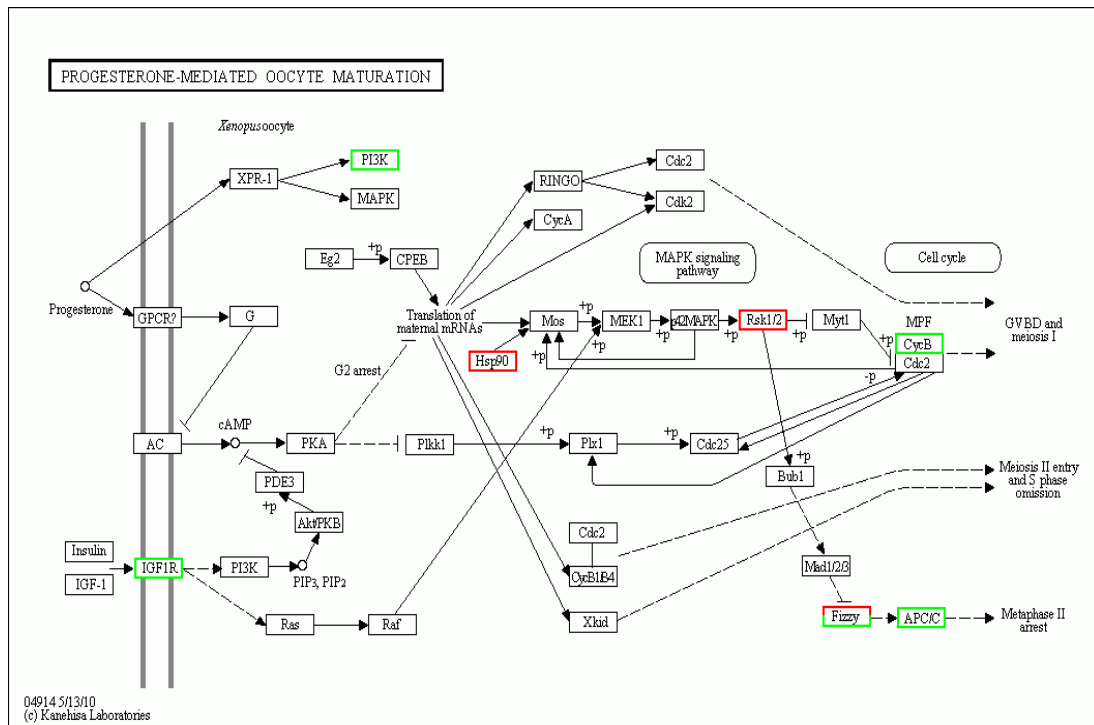

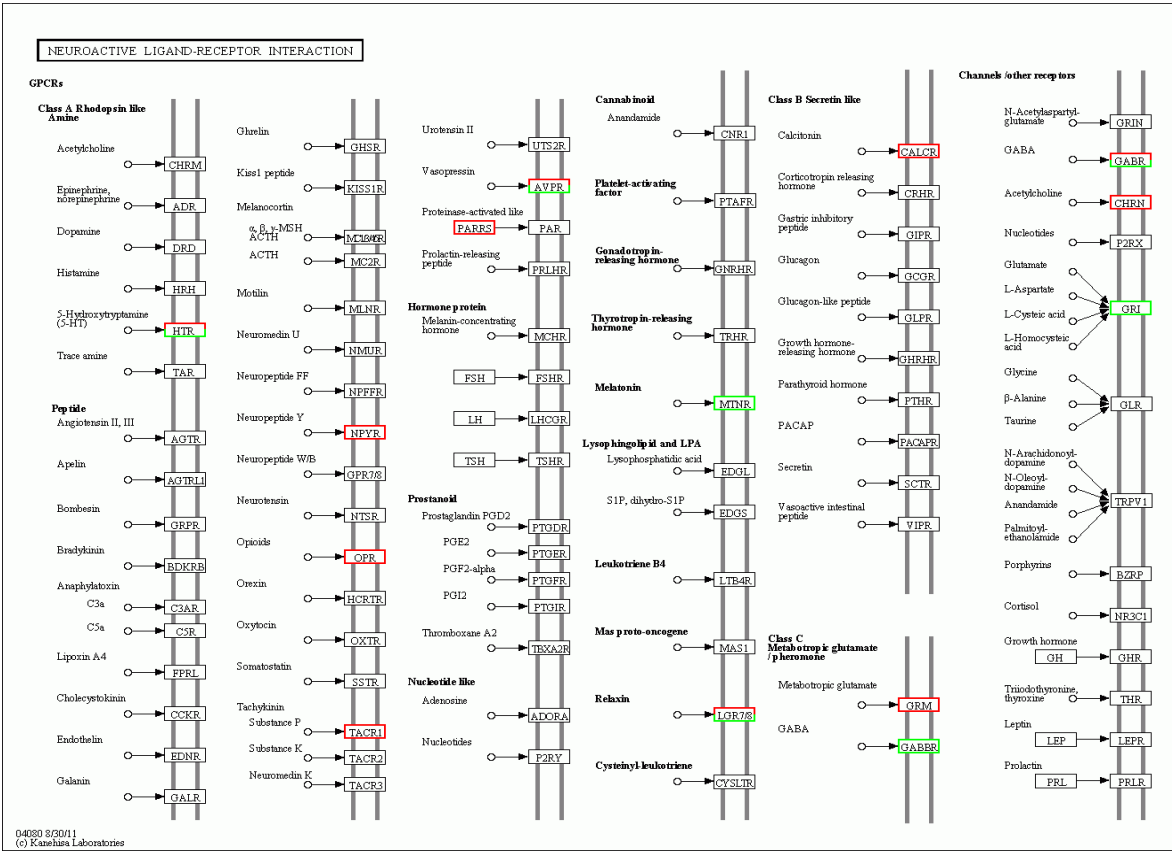

Supplement: Supplementary file 1 — Figure S1: COG function classification of Eriocheir sinensis androgenic gland transcriptome. Figure S2: Differentially regulated genes involved in insulin signaling pathway. The up-regulated and down-regulated genes are labeled in red and green, respectively. Figure S3: Differentially regulated genes involved in progesterone-mediated oocyte maturation. The up-regulated and down-regulated genes are labeled in red and green, respectively. [file 4956216.f1.pdf]
